# Intracellular targets of RGDS peptide in melanoma cells

**DOI:** 10.1186/1476-4598-9-84

**Published:** 2010-04-22

**Authors:** Maria Simona Aguzzi, Paola Fortugno, Claudia Giampietri, Gianluca Ragone, Maurizio C Capogrossi, Antonio Facchiano

**Affiliations:** 1 Laboratorio Patologia Vascolare, Istituto Dermopatico dell'Immacolata, IDI-IRCCS, Roma, Italy; 2 Laboratorio Biologia Molecolare e Cellulare, Istituto Dermopatico dell'Immacolata, IDI-IRCCS, Roma, Italy; 3 Dipartimento di Istologia e Embriologia Medica, Università di Roma "Sapienza", Roma, Italy; 4 Laboratorio Oncogenesi Molecolare, Istituto Dermopatico dell'Immacolata, IDI-IRCCS, Roma, Italy

## Abstract

**Background:**

RGD-motif acts as a specific integrins-ligand and regulates a variety of cell-functions via extracellular action affecting cell-adhesion properties. However, increasing evidence identifies additional RGDS-functions at intracellular level. Previous reports show RGDS-internalization in endothelial cells, cardiomyocytes and lymphocytes, indicating intracellular targets such as caspase-8 and caspase-9, and suggest RGDS specific activity at cytoplasmic level. Given the role RGDS-peptides play in controlling proliferation and apoptosis in several cell types, investigating intracellular targets of RGDS in melanoma cells may un-reveal novel molecular targets and key pathways, potentially useful for a more effective approach to melanoma treatment.

**Results:**

In the present study we show for the first time that RGDS-peptide is internalized in melanoma cells in a time-dependent way and exerts strong anti-proliferative and pro-apoptotic effects independently from its extracellular anti-adhesive action. RGES control-peptide did not show biological effects, as expected; nevertheless it is internalized, although with slower kinetics. Survivin, a known cell-cycle and survival-regulator is highly expressed in melanoma cells. Co-immunoprecipitation assays in cell lysates and overlay assays with the purified proteins showed that RGDS interacts with survivin, as well as with procaspase-3, -8 and -9. RGDS-peptide binding to survivin was found to be specific, at high affinity (Kd 27.5 μM) and located at the survivin C-terminus. RGDS-survivin interaction appeared to play a key role, since RGDS lost its anti-mitogenic effect in survivin-deprived cells with a specific siRNA.

**Conclusions:**

RGDS inhibits melanoma growth with an adhesion-independent mechanism; it is internalized in melanoma cells and specifically interacts with survivin. The present data may indicate a novel role of RGDS-containing peptides physiologically released from the extracellular matrix and may suggest a possible novel anti-proliferation strategy in melanoma.

## Background

RGD (Arg-Gly-Asp) motif is largely investigated as mediator of cell adhesion to extracellular matrix and to cells, via cell-surface receptors named integrins. These receptors belong to a large family of twenty-four heterodimeric members. Several integrins, including α_v_β_3_, α_5_β_1_, α_v_β_5_, α_v_β_6 _and α_IIb_β_3, _recognize the RGD motif present in various ECM proteins such as fibronectin, vitronectin, laminin, fibrinogen, von Willebrand factor, osteopontin, thrombospondin, and collagen [1] as well as in disintegrins; others including α_1_β_1_, α_2_β_1_, α_10_β_1_, and α_11_β_1_, interact with the matrix in a RGD-independent manner [2-4]. Integrins activation triggers different signals regulating cell adhesion, migration, survival, apoptosis [1,5,6] as well as processes such as angiogenesis, thrombosis and osteoporosis [7-9]. Further, integrins control the interaction of tumor cells with the surrounding environment, with a direct effect on cell proliferation, migration, metastatic dissemination, invasion, cell survival [10,11]. RGD motif-containing peptides bind integrin receptors with high affinity and inhibit cell adhesion by competing the integrins/matrix interaction leading to anti-inflammatory, anti-coagulant and anti-metastatic effects, as well as anti-angiogenic effects [12-15]. RGD peptides are also involved in tumor and endothelial cells-targeting via the α_v_β_3 _receptors [16-19] as well as in noninvasive tumor imaging, targeting and radio-treatment [20].

RGD-containing peptides inhibit cell growth by inducing cells-detachment from extracellular matrix and adhesion-dependent apoptosis, named anoikis [21]. An additional mechanism, previously investigated by us and Others, involves RGD peptides cell-internalization, intracellular targeting and direct activation of caspase-3, caspase-8 or caspase-9 in lymphocytes, cardiomyocytes, endothelial cells and chondrocytes, leading to apoptosis most-likely via an integrin-independent mechanism [3,22-24]. These findings suggest that RGD motif, in addition to targets exposed onto the external surface of cell membrane, recognizes intracellular targets (namely caspases), leading to procaspase auto-processing and activation.

Survivin is a member of the Inhibitor of Apoptosis Protein (IAPs) family; it is involved in multiple functions, including control of cell division, apoptosis and cellular response to stress [25]. Survivin is selectively expressed during development and in proliferating cells; it increases during G1 cell-phase and reaches a peak-level at G2-M phase. Survivin expression level is very low or undetectable in most differentiated tissues, in the absence of stress conditions, while normally it is expressed in thymus, basal colonic epithelium, endothelial cells and neural stem cells during angiogenesis [25]. Survivin plays a critical role in cancer biology; it is selectively expressed in transformed cells and in most cancers as breast, lung, pancreatic and colon carcinomas, haematological tumors, sarcomas and neuroblastoma. Further, it is expressed in melanocytic nevi, melanoma metastatic lesions and invasive melanomas, but not in normal melanocytes [26]. Survivin is a survival factor for cancer cells and its over-expression correlates with unfavorable prognosis, high recurrence risk, metastasis, high resistance to both chemo- and radio-therapy [27]. Due to its role in cancer resistance to apoptotic stimuli, survivin has been proposed as a potential target for anticancer therapies based on antisense oligonucleotides, small interfering RNAs, ribozymes and dominant negative mutants [28-31].

In the present study we investigated for the first time intracellular targets of RGDS peptide in human metastatic melanoma cells and identified survivin as a novel direct target of RGDS molecule.

## Results

### RGDS effect on SK-MEL-110 proliferation and apoptosis

**Figure 1:**
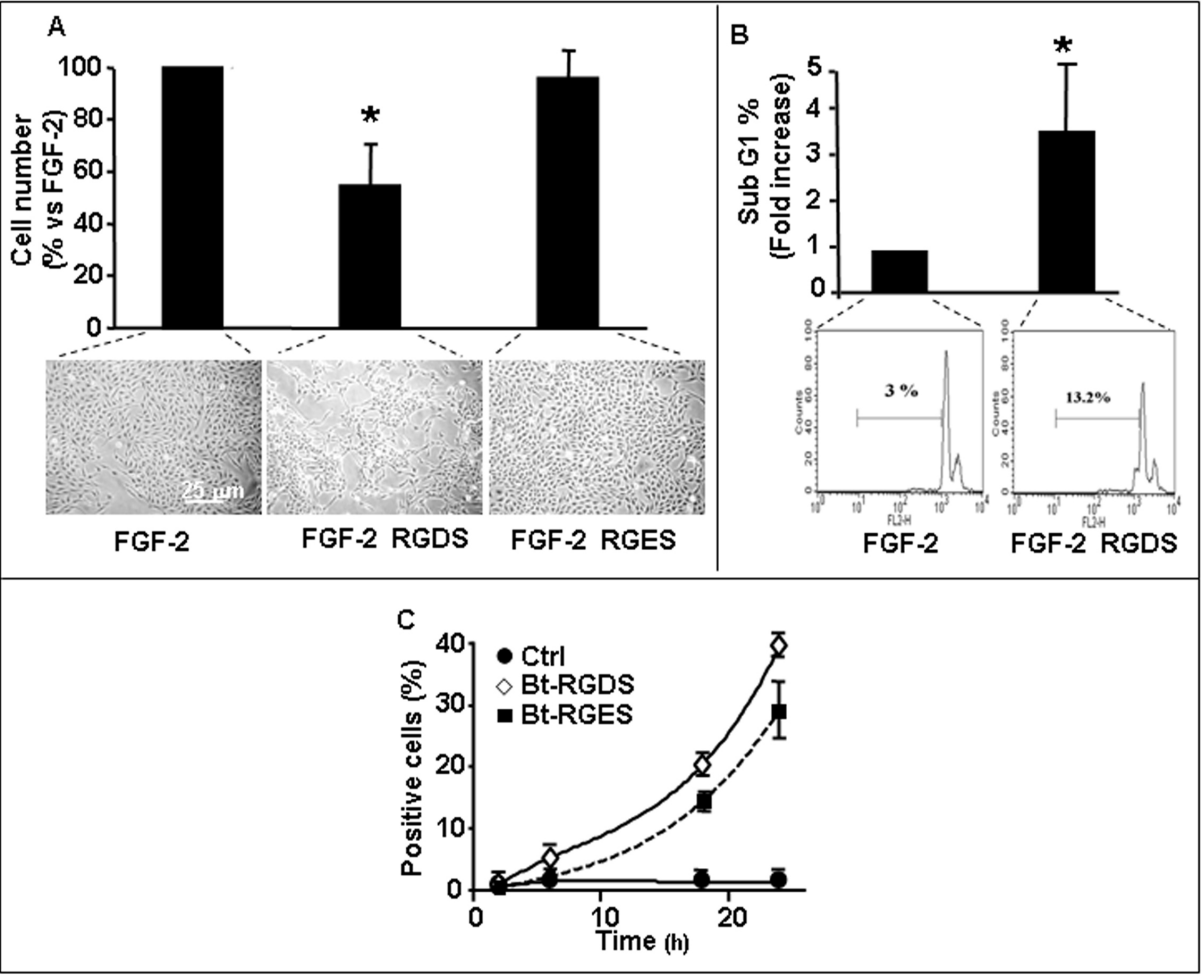
**SK-MEL-110 proliferation and apoptosis**. **A: **SK-MEL-110 seeded on collagen-IV (50 μg/ml) were treated for 48 h with RGDS or RGES (500 μg/ml) in the presence of FGF-2 (10 ng/ml). Data are expressed as mean ± SD of 5 experiments carried out in duplicate. Representative images are reported. **B: **SK-MEL-110 apoptosis was quantified by FACS analysis of PI-stained cells after 48 h RGDS treatment (500 μg/ml) (p < 0.05 *vs *FGF-2). Three independent experiments were performed and quantified; one representative experiment is shown. **C**: Biotinylated-RGDS and RGES internalization in SK-MEL-110 was measured by FACS. Cells were treated for different time points (2, 6, 16 and 24 h) with bt-RGDS or bt-RGES; internalization was revealed by PE-avidin. Three independent experiments were performed.

As we and others previously demonstrated [2-4], cell adhesion to collagen-IV is RGD-independent. We further confirmed such observation on SK-MEL-110 cells (unpublished data). We therefore investigated cells seeded onto collagen IV-coated plastic throughout the whole study, in order to investigate RGDS-effects independently of its anti-adhesive action. Melanoma cells proliferation induced by FGF-2 was significantly reduced in the presence of RGDS (Figure 1A top) (46 ± 16% inhibition, p < 0.005) at 500 μg/ml, indicating that, despite the presence of the strong survival factor FGF-2, RGDS exerts a potent anti-proliferative effect on SK-MEL-110 cells, independently of its anti-adhesive properties. Control peptide RGES had no anti-proliferative effect, as expected. The effect is shown in one representative field (Figure 1A bottom).

RGDS-treated cells were stained with propidium iodide (PI) and cell cycle was investigated. RGDS-treatment (48 h, 500 μg/ml) increased the percentage of cells in sub-G1-phase from 3% (with FGF-2 alone) to 13.2% (with FGF-2 and RGDS) (Figure 1B top and bottom), indicating that RGDS may induce apoptosis in melanoma cells, in the presence of FGF-2. RGDS-treatment also increased number of cells in G1-phase, *vs *control (77% vs 67%), and markedly reduced cell-number in S (3.5% *vs *8.7%) and in G2-phase (9% *vs *16%) (Additional file 1).

### RGDS cell internalization

**Figure 2:**
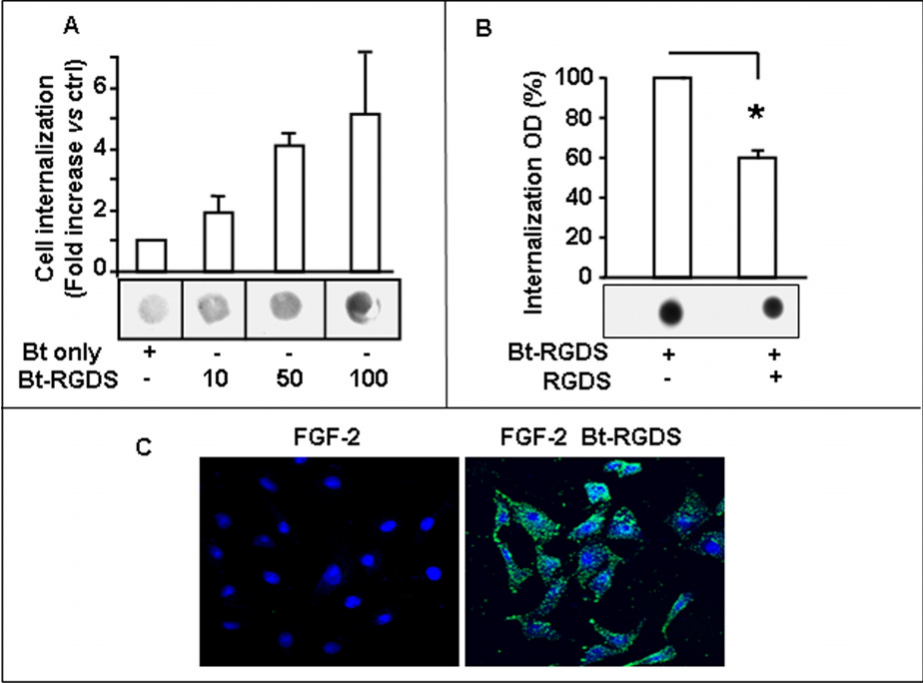
**RGDS internalization in melanoma cells**. **A: **Bt-RGDS internalization into live SK-MEL-110 was examined after 24 h incubation at 37°C. Cells were treated with biotin alone or with increasing concentrations of bt-RGDS, cytoplasmic extracts were immobilized onto nitrocellulose and biotin presence was detected by avidin-peroxidase kit. Densitometry-quantification of total internalization in three experiments and one representative experiment are reported. **B: **Total internalization measured in extracts obtained from cells treated with bt-RGDS (50 μg/ml), and nonspecific internalization in the presence of an excess of unlabeled RGDS (1 mg/ml). Densitometry of three separate experiments and one representative experiment are reported. **C: **Bt-RGDS internalization (500 μg/ml) at 24 h was confirmed by confocal microscopy (original magnification × 40). RGDS entered into live melanoma cells (green stain) with a prevalently cytoplasmic localization. Nuclei are shown as blue stain. This experiment was carried out three times in duplicate.

We then investigated whether RGDS is internalized into SK-MEL-110, by exploiting different experimental approaches. RGDS internalization in live cells was quantified by FACS analysis. The biotinylated peptide entered into melanoma cells in a time-dependent manner (Figure 1C); positive cells reached about 40% of total cells at 24 h incubation. RGES internalized with slower kinetics, reaching about 30% of positive cells at 24 h (i.e., 25% less than RGDS). RGDS internalization was markedly higher in melanoma cells *vs *HUVEC (Additional file 2). RGDS internalization was also measured in cytoplasmic extracts of SK-MEL-110 exposed to increasing doses of bt-RGDS for 24 h at 37°C or biotin alone as control (Figure 2A). Internalization was measured by densitometry and was inhibited by an excess of not-biotinylated RGDS, indicating a specific internalization accounting for about 60% of the total internalization (Figure 2B). Confocal microscopy further supported these results, indicating a mostly cytoplasmic intracellular localization (Figure 2C) at 24 h treatment.

### RGDS-survivin interaction

**Figure 3:**
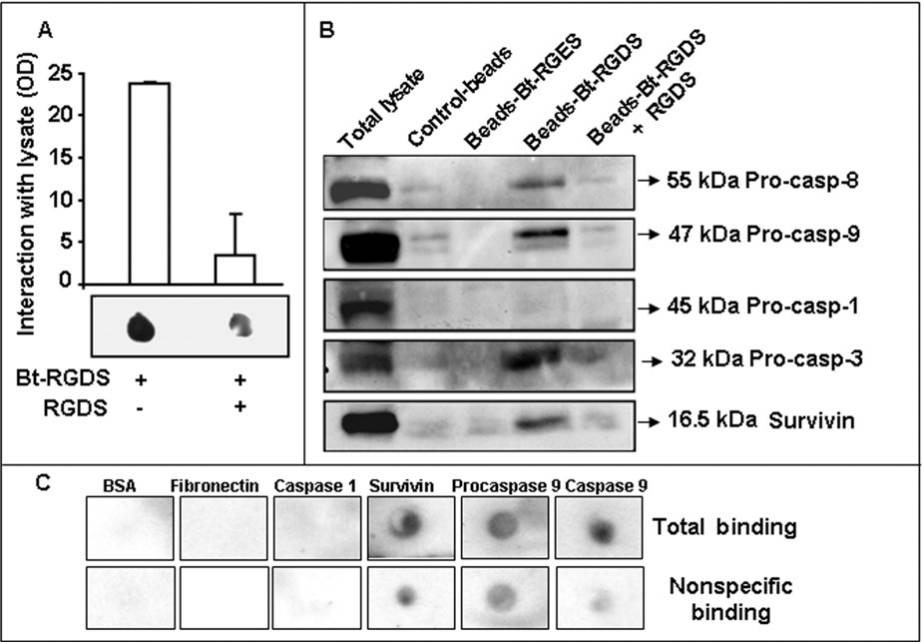
**RGDS-survivin interaction. A: **Bt-RGDS interaction with cell lysate: cytoplasmic extracts were immobilized onto nitrocellulose and incubated with bt-RGDS. Total binding in the presence of labeled RGDS and nonspecific binding in the presence of an excess of unlabeled RGDS are shown. Densitometry of three different experiments and one representative experiment are reported. **B: **RGDS interaction with intracellular proteins was assayed by co-precipitation. BSA (control), bt-RGES (specificity control) or bt-RGDS (1 mM) were incubated for 1 h at 4°C with streptavidin-coated dynabeads, then SK-MEL-110 lysate, pre-incubated for 4 h at 4°C with an excess of unlabeled RGDS, was added and incubated overnight at 4°C. Precipitated proteins were revealed by western blotting using various antibodies (anti pro-caspase-8, anti pro-caspase-9, anti pro-caspase-1, anti pro-caspase-3 and anti-survivin). Total lysate was used as positive control. This experiment was carried out three times. **C: **Purified recombinant proteins were spotted onto nitrocellulose (0.9 μg/spot). Membrane was incubated for 4 h at RT with bt-RGDS (1 mg/ml) (total binding) in the absence or in the presence of an excess of unlabeled RGDS (10 mg/ml) (nonspecific binding). Three independent experiments were performed.

The observation that RGDS is able to internalize into melanoma cells raised the question whether RGDS may recognize intracellular targets. Biotinylated-RGDS (bt-RGDS) was found to directly interact with cytoplasmic extracts and such binding was strongly inhibited by an excess of unlabeled-RGDS (Figure 3A), suggesting that RGDS recognizes specific targets in cytoplasmic lysates.

Survivin is known to be highly expressed in tumors and melanoma playing a key role in survival control [32,33]. In SK-MEL-110 melanoma cells it is expressed at levels higher than endothelial cells (HUVEC) or melanocytes (Additional file 3).

**Figure 4:**
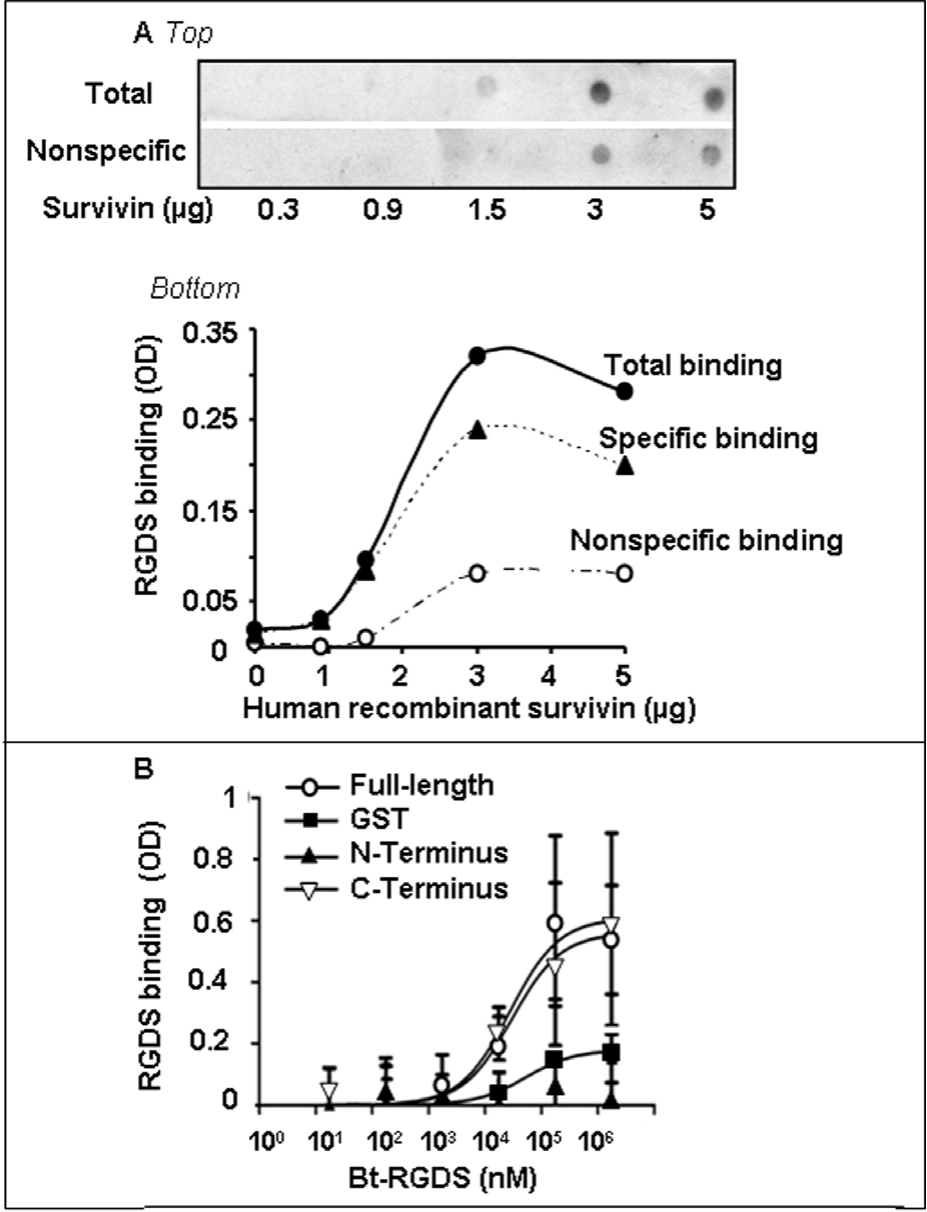
**RGDS-survivin binding. A: **Increasing doses of purified human-recombinant survivin (0.3 to 5 μg/spot) were immobilized onto membrane and incubated with bt-RGDS in the presence of un-labeled peptide, as competitor (*top*). A representative experiment of two different assays, is shown. Densitometry quantification is reported (*bottom*). **B: **Bt-RGDS binding to recombinant GST-fusion survivin and to N-terminus (Met1-Gly99) or C-terminus (Lys90-Asp142) fragments (175 mM) was evaluated by solid-phase assay (SPA). Proteins were immobilized onto plastic and were incubated with increasing doses of bt-RGDS (1.75 nM to 1.75 mM) in the presence of an excess of un-labeled RGDS. Three independent experiments were performed.

We thus hypothesized a functional interaction between survivin and intracellular RGDS. RGD peptides have been reported by us and by Others to interact with intracellular targets such as pro-caspase-3, caspase-8 and caspase-9 in other cell types [3,22,23]. We hypothesized that RGDS may recognize additional intracellular targets, namely survivin. Cytoplasmic lysates of growing SK-MEL-110, were incubated with bt-RGDS-coated dynabeads. Proteins interacting with RGDS were then revealed by SDS-page and western blotting and were identified with specific detecting-antibodies, as pro-caspase-8, pro-caspase-9, pro-caspase-3 and survivin, while RGDS did not interact with pro-caspase-1. Pre-incubating lysates with not-biotinylated RGDS abolished interaction of bt-RGDS with survivin, caspase-3, caspase-8 and caspase-9, indicating a specific binding; such conclusion was further supported by the observation that bt-RGES control peptide is not able to bind with any tested intracellular protein (Figure 3B). Direct binding of RGDS with intracellular proteins was further investigated in overlay assays by direct immobilization of several different proteins onto nitrocellulose membrane. Labeled RGDS was found to directly interact with precursor- and active-caspase-9 and with recombinant survivin and this binding was specifically inhibited, at least in part, in the presence of an excess of unlabeled RGDS. BSA, fibronectin or active caspase-1 did not interact with RGDS (Figure 3C). To further investigate such interaction, increasing doses of recombinant survivin (0.3 to 5 μg) were immobilized onto nitrocellulose membrane and incubated with the bt-RGDS. RGDS bound survivin in a concentration-dependent and saturable manner. Specific binding was computed by subtracting nonspecific binding from the total binding (Figure 4A top and bottom). As a further approach, a solid phase assay was carried out. Recombinant GST (as control), GST-Full length survivin, GST-N-terminus-survivin and GST-C-terminus-survivin were immobilized onto plastic and incubated with increasing concentrations of bt-RGDS in the presence or in the absence of an excess of unlabeled RGDS. Under such conditions RGDS bound Full-length survivin and C-Terminus-survivin in a dose-dependent and saturable way. A Kd of 27.5 μM and 30 μM, respectively and a Bmax of 0.61 and 0.56, for the binding to Full-length and C-terminus, respectively (Figure 4B), were computed according to the one-site binding curve-fit equation (1). In contrast, RGDS did not bind the GST-N-terminus fragment (Kd of -161 and Bmax of -0.0079). These data suggested that RGDS-survivin interaction occurs at a single site at the C-Terminus region of survivin.

### Survivin small interference-RNA

**Figure 5:**
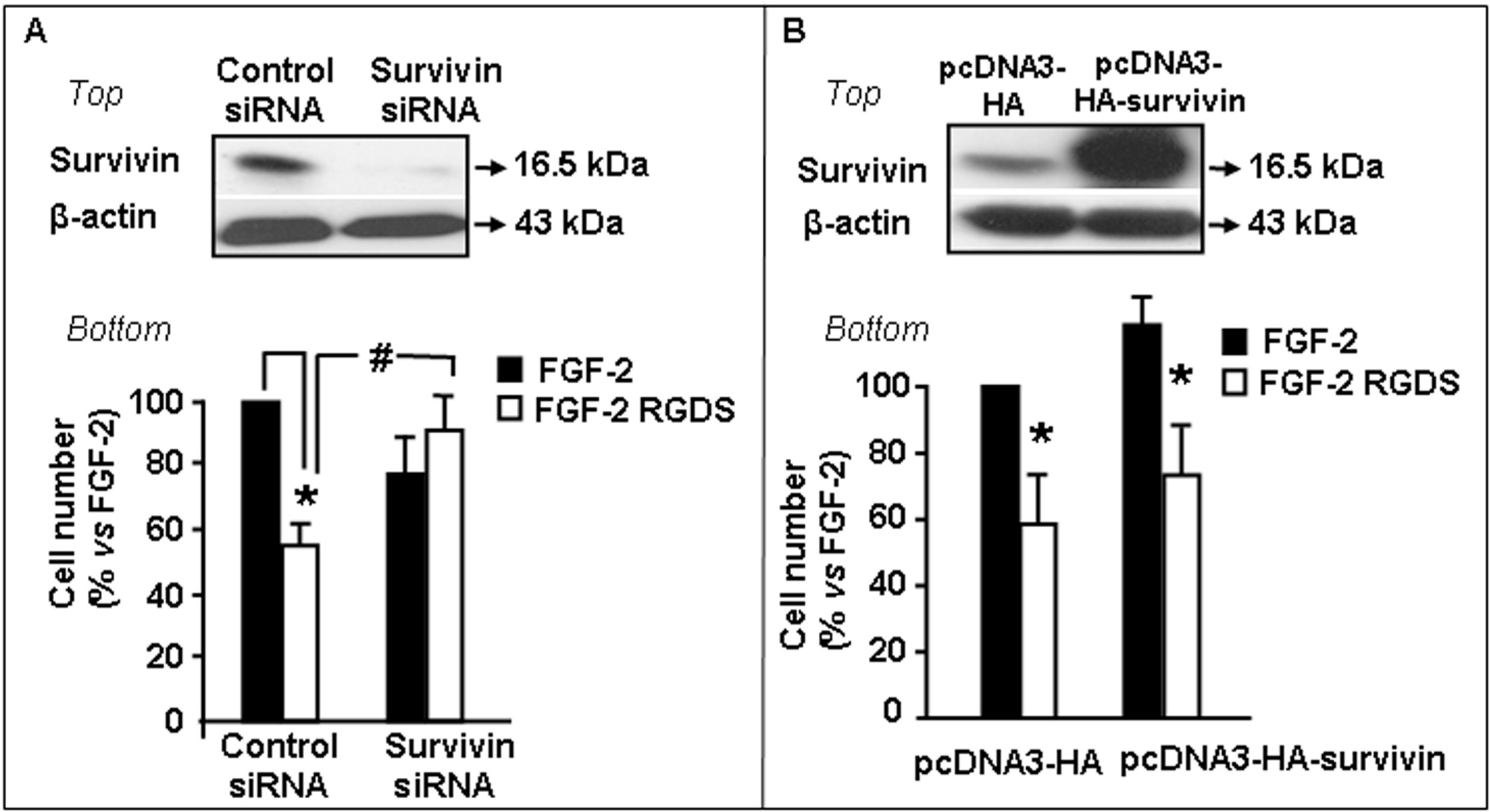
**RGDS effect require survivin expression**. SK-MEL-110 were transfected with human survivin siRNA or with control siRNA before RGDS treatment. SiGLO Lamin A/C siRNA was used as control of transfection efficiency. **A ***Top***: **Survivin silencing was confirmed by western blotting after 72 h transfection. *Bottom***: **FGF-2 (10 ng/ml)-induced proliferation in survivin-silenced or in control, after RGDS treatment for 48 h (* p < 0.05 *vs *FGF-2 and # p < 0.005 vs FGF-2 in silenced cells). Three independent experiments were performed in duplicate. **B ***Top***: **Full length HA-tagged survivin was over-expressed in melanoma cells before RGDS treatment. Over-expression was confirmed by western blotting after 24 h transfection. *Bottom***: **RGDS effect on FGF-2-induced proliferation in transfected-SK-MEL-110 after 48 h treatment (p < 0.01 *vs *FGF-2). Three independent experiments were performed in duplicate.

The reported results demonstrate a specific interaction of RGDS and survivin. We then investigated whether survivin mediates RGDS effects on cell proliferation/survival. To this aim survivin was silenced in SK-MEL-110 and cells were treated with RGDS for 48 h. SiGLO Lamin A/C siRNA was used as positive control of transfection efficiency. Survivin silencing was confirmed by western blotting and showed complete protein down-regulation (Figure 5A top) and induced high mortality in SK-MEL-110 (35%, data not shown), as expected. Under such survivin-deprivation conditions, FGF-2 has a reduced survival effect, as expected, and RGDS completely looses its inhibitory effect (Figure 5A bottom), suggesting that the RGDS anti-proliferative action requires, at least in part, survivin presence. To further confirm such hypothesis, the opposite approach was followed, and survivin-forced expression was achieved by transfection with a specific expression plasmid. Western blotting analysis confirms survivin overexpression as compared to the control (Figure 5B top); under such experimental conditions FGF-2 survival effect is increased, as expected, and RGDS maintains its anti-mitogenic effect (Figure 5B bottom).

### RGDS effect on caspase-3 expression

**Figure 6:**
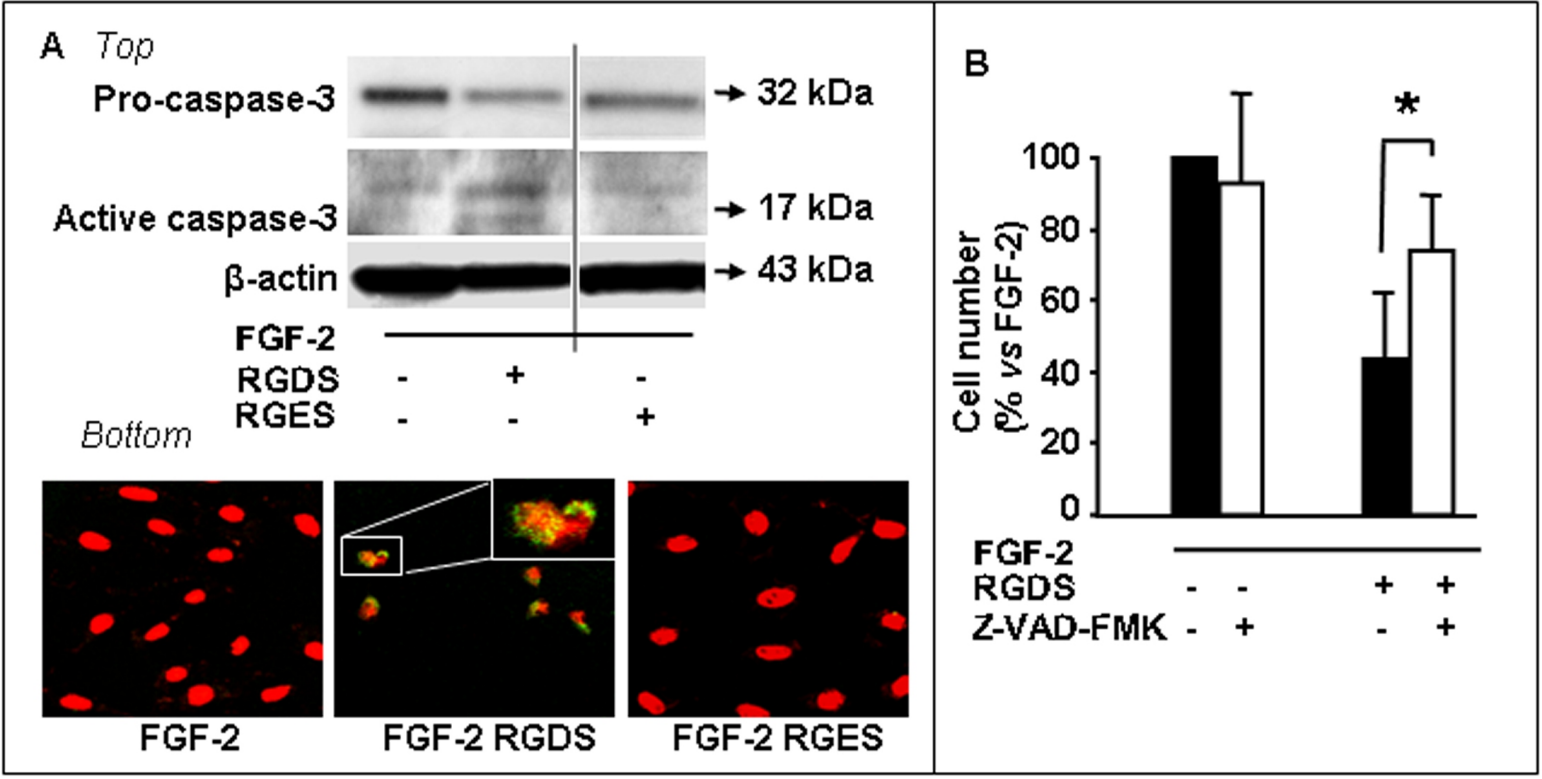
**RGDS treatment activates caspase-3**. **A: **SK-MEL-110 seeded on collagen-IV were incubated for 48 h with FGF-2 in the presence or in the absence of RGDS or RGES (500 μg/ml). T*op: *Pro-caspase 3 cleavage and caspase-3 activation were observed by western blotting. *Bottom: *caspase-3 activation was confirmed by confocal microscopy using an anti-active caspase-3 antibody. **B: **Pre-treatment (2 h) of melanoma cells with Z-VAD-FMK (50 μM), a general caspases inhibitor, abolished the RGDS anti-proliferative effect (p < 0.005 vs FGF-2 in the presence of Z-VAD-FMK). All these experiments were carried out three times in duplicate.

We and Others previously demonstrated that RGDS activates caspase-3 in different cell types [3,22,23], leading to apoptosis. In the present study 48 h RGDS treatment reduced pro-caspase-3 (molecular weight 32 kDa) and increased expression of caspase-3 active subunit (17 kDa) in SK-MEL-110 (Figure 6A top). Caspase-3 activation was confirmed by confocal microscopy using a specific antibody able to recognize the active form, while RGES control peptide had no effect on caspase-3 activation (Figure 6A bottom). Pre-treatment of melanoma cells for 2 h with Z-VAD-FMK, a general caspases inhibitor, significantly abolished the RGDS anti-proliferative effect (Figure 6B), further indicating that RGDS action was caspase-dependent.

## Discussion

The RGD motif occurs in several ECM proteins and plays a crucial role in integrin-mediated cell adhesion. It has been largely investigated in the past decades as mediator of integrins-dependent cell-adhesion and, consequently, for the effect on survival, invasion, blood coagulation [21,34,35]. Recent evidence by different investigators has identified a novel role, namely the RGD ability to act at the intracellular level, to recognize intracellular targets and to activate the apoptotic cascade via a direct caspase-activation process and caspase cleavage [3,22-24]. Thus a possible role of RGD-containing peptides released from the extracellular matrix and accumulated in the cytosolic compartment has been suggested [36], highlighting the view that extracellular matrix degradation occurring during physiologic and pathologic tissue-remodeling, may generate RGD-containing products acting at the intracellular-level.

We have previously demonstrated that under experimental conditions most-likely abrogating extracellular anti-adhesive effect, RGDS induces apoptosis in HUVEC in integrins-independent way and directly interacts with caspase-8 and caspase-9 in HUVEC [3]. RGDS peptide has been studied in several melanoma setting, mostly as an anti-adhesion molecule [37-39], while no data are available on the intracellular effect of RGDS in melanoma cells. Hence we addressed RGDS internalization in these cells and investigated novel intracellular targets involved in the control of melanoma cells survival. Cell adhesion to collagens is RGD-independent [2-4]; thus, in cells seeded onto collagen-IV most of the RGDS biological effects do not depend on its anti-adhesive effects. Under such conditions, RGDS significantly inhibited proliferation of SK-MEL-110 in the presence of FGF-2 and induced apoptosis, while the control-peptide RGES was ineffective. RGDS peptide was internalized into live melanoma cells and specifically interacted with the cytoplasmic extract. RGDS-internalization and anti-mitogenic/pro-apoptotic effects were observed in melanoma cells (in the present study) and in HUVEC [3], although kinetics and potency are different due to the different intrinsic features of these two cell types. Interestingly, RGES peptide did not show biological effects but was internalized, although with slower kinetics compared to RGDS. This observation agrees with previous data collected on other RGDS controls [23] and opens the question regarding mechanisms of internalization and its specificity. Further investigations are needed to discriminate between passive diffusion of small molecules such as RGES and RGDS, and specific-RGDS-internalization [40]. RGDS directly and specifically bound pro-caspase-8, pro-caspase-9 and pro-caspase-3, while it did not bind pro-caspase-1, a pro-inflammatory caspase not directly involved in the apoptosis cascade. Such data may support the hypothesis that intracellular targets of RGDS may comprise different molecules of the apoptotic cascade. Conversely, RGES did not interact with any tested intracellular target. Survivin was found to be a novel RGDS intracellular direct target and a specific RGDS-binding site was found at survivin C-terminus, where an extended alpha-helical coiled-coil portion is located. Such domain is known to play a key role for the survivin interaction with microtubules and cell division-control. Additional studies will be carried out to identify the specific amino acids involved in RGDS-survivin interaction. Survivin controls cell division, apoptosis and cellular stress response and protects cells against both caspase-dependent and caspase-independent cell death [41,42]. Its interaction with RGDS although un-expected, was not surprising, given the previous evidence showing direct interaction of RGDS with other key players of the survival/apoptosis machine [3,22,23]. Survivin-silencing achieved with a specific siRNA suppressed the anti-mitogenic action of RGDS, while the anti-proliferative effect of RGDS was maintained when survivin expression was up-regulated with a specific expression plasmid, indicating that survivin is required for RGDS anti-mitogenic effect in SK-MEL-110 and that cell-sensitivity to RGDS actions may depend at least in part on survivin levels in different physiological and pathological conditions. Such consideration supports survivin as a potential target for anti-tumor approaches. Indeed, several studies demonstrated that anticancer drugs as colecoxib, COX-2 inhibitors or silibilin derivates down-regulate survivin expression in a wide range of tumor cells, inducing apoptosis [43,44].

## Conclusions

The present study indicates that in melanoma cells RGDS peptide interacts with intracellular targets, namely apoptotic caspases and survivin; identifies RGDS as a survivin-targeting molecule and indicates a novel mechanism to control cell proliferation.

## Methods

### Cells culture

Human metastatic melanoma cells line SK-MEL-110 [45] were grown in DMEM (Hyclone, Logan, UT) supplemented with 2 mM L-glutamine, 100 IU/ml penicillin-streptomycin (Gibco, Invitrogen corporation, Carlsbad, CA), and 10% heat-inactivated FCS (Hyclone, Logan, UT). Cells were cultured at 37°C in a 5% CO_2 _atmosphere.

### Proliferation assay and apoptosis

SK-MEL-110 were plated in 6-well plates (8 × 10^4 ^cells/well) on collagen-IV (50 μg/ml) and were grown for 24 h in complete medium in 5% CO_2. _Cells were then serum-starved for 24 h and subsequently treated with RGDS (Bachem, Bubendorf, Switzerland) or RGES as control (Sigma-Aldrich, St Louis, MO) (500 μg/ml) dissolved in DMEM containing FGF-2 (Pierce-Endogen, Rockford, IL) for 48 h. Then, cells were photographed, harvested by trypsin-EDTA and counted using hemacytometer. In other experiments, cells were pre-treated with a general caspase inhibitor (Z-VAD-FMK) (50 μM) (R&D Systems, Minneapolis, MN) for 2 h before RGDS treatment. To analyze cell cycle and sub-G1-phase, cells were fixed in ice-cold 70% ethanol and stained with propidium iodide (PI) at 10 μg/ml final concentration. Flow cytometry was performed on a Profile I flow cytometer (FACSCalibur, BD-Biosciences).

### Biotinylated-RGDS (bt-RGDS) internalization and interaction with recombinant proteins

To investigate peptides internalization, cells were treated with the biotinylated-RGDS (bt-RGDS) or biotinylated-RGES (bt-RGES) (NeoMPS-SA, Strasbourg, France) as control for different time-points, stained with phycoerythrin conjugated-avidin and analyzed by FACS. In additional experiments cells grown on collagen-IV were serum-starved for 48 h and treated for 24 h with biotin alone or with different doses of bt-RGDS (10-50-100 μg/ml). In other experiments cells were treated with 50 μg/ml bt-RGDS with an excess of 1 mg/ml un-labeled RGDS as specific competitor. Cells were washed to eliminate bt-RGDS bound to the membranes and a cytoplasmic extract was prepared as previously reported [3]. Cytoplasmic lysates were spotted onto nitrocellulose, blocked with 5% milk in TPBS (0.1% Tween 20 in PBS) and incubated for 1 h at RT with a Vectastain ABC-peroxidase kit (Vector) followed by chemiluminescence reaction and exposure to Kodak film (Eastman Kodak). Interaction was quantified by densitometry (GS 710; Bio-Rad) and analyzed using the "Quantity one" software (Bio-Rad).

In other experiments 40 μg of growing cells cytoplasmic extract, or increasing doses of recombinant human survivin (0.3-0.9-1.5-3-5 μg) (CP-Biotech, Sylvania, OH) or other recombinant proteins, as caspases-1 and -9, pro-caspase-9 (Alexis), fibronectin (0.9 μg) (Becton-Dickinson, Bradford, MA) and BSA were spotted onto nitrocellulose, incubated for 4 h at RT with bt-RGDS (1 mg/ml) in the presence or absence of RGDS-excess (10 mg/ml) to measure the specific binding.

### Confocal microscopy

SK-MEL-110 seeded on coverslips coated with collagen-IV and treated for 24 h with bt-RGDS (500 μg/ml), were washed as previously reported to eliminate the peptide bound to the membranes [3]. Cells were fixed with 3% paraformaldehyde in PBS, pH 7.4, for 10 minutes, permeabilized with 0.1% Triton X-100 in PBS, pH 7.4, for 5 minutes at RT, and blocked for 30 minutes with BSA 2% in PBS, pH 7.4, at RT, followed by incubation with fluorescein avidin (1:40; Vector-Laboratories, Peterborough, UK) in PBS, pH 7.4, for 1 h at RT. After washing in 0.3% Triton X-100 in PBS, cells were incubated with PI at a final concentration of 5 μg/ml to visualize nuclei and analyzed using a Zeiss LSM 510 meta-confocal microscope (Zeiss). Laser power, beam splitters, filter settings, pinhole diameters and scan-mode were the same for all examined samples. To visualize active caspase-3 form, cells were treated with RGDS or RGES, fixed and incubated with a rabbit-monoclonal anti-active caspase-3 (1:150) (Abcam Inc,. Cambridge, MA).

### Western blotting

SK-MEL-110 treated as described in the proliferation assay paragraph, were lysed with RIPA buffer. Samples were boiled, loaded and separated by sodium dodecyl sulfate-polyacrylamide gel electrophoresis (SDS-PAGE) and transferred to nitrocellulose membrane. Membrane was blocked with 5% milk (Bio-Rad Laboratories) in TPBS (0.1% Tween 20 in PBS, pH 7.4), washed and incubated with mouse anti-survivin (1:200) (Santa Cruz Biotechnology, Santa Cruz, CA), rabbit anti-caspase 3 (1:200) (Santa Cruz Biotechnology, Alexa, CA,), or mouse anti-β-actin (1:5000) (Sigma-Aldrich, St Louis, MO) in milk 5% TPBS 0.1% for 1 h at RT. Horseradish peroxidase-conjugated secondary antibodies (Pierce) were used, followed by chemiluminescence (ECL; Amersham, Buckinghamshire, United Kingdom) and autoradiography.

### Precipitation with streptavidin-coated Dynabeads

Streptavidin-coated Dynabeads (M-270, 2.8 μm, Dynal) were re-suspended, washed in PBS three times, using a magnetic holder, re-suspended and 1 × 10^3 ^pmoles of bt-RGDS, BSA or bt-RGES per mg beads were added, incubated for 1 h at 4°C and washed in PBS for five times. Growing SK-MEL-110 were lysed as reported [22], pre-incubated with 10 μl RGDS as specific competitor (1 mM) and incubated with activated beads, at 4°C overnight with unidirectional mixing. Samples were boiled and separated by SDS-PAGE as described above and incubated with antibody to survivin (1:200), caspase-3 (1:200), caspase-8 (1:200) (Santa Cruz Biotechnology, Alexa, CA), caspase-9 (1:1000) (Pharmingen, San Diego, CA), caspase-1 (1:500) (Alexis, San Diego, CA). Detection was performed as described above.

### Survivin cloning and expression

Full length HA-tagged Survivin cDNA (GeneBank NM_001168) was generated by PCR using total cDNA from HeLa cells as template.

The primers used were:

GATCAAGCTTATGTATCCGTATGATGTTCCTGATTATGCTGGTGCCCCGACGTTGCC (forward) and GATCGGATCCGGAAGTGGTGCAGCCACTC (reverse) (Invitrogen).

Restriction sites (underlined) for the endonucleases HindIII and BamHI were used for cloning in pcDNA3 vector (Invitrogen). All constructs made by PCR were sequence-verified. Recombinant HA-survivin was expressed in SK-MEL-110 by transient transfection using Lipofectamine Plus Reagent (Invitrogen) according to the manufacturer's instruction. pcDNA-HA empty vector was used as control. Transfection efficiency was evaluated using a pEGFP-N1 reporter vector at a HA-survivin plasmid molar ratio of 4:1. FGF-2-induced proliferation in transfected cells was evaluated after RGDS 48 h treatment. Protein expression was detected by western blotting.

Full length survivin and both N-terminus (Met1-Gly99) or C-terminus (Lys90-Asp142) were PCR amplified and cloned, using EcoRI and BamHI restriction sites, in pGex6P2 (GE Healthcare) for expression of GST fusion proteins in *E. coli *BL21(DE3)pLysS (PROMEGA, Southampton, United Kingdom).

The primers used were:

GAT***GGATCC***ATGGGTGCCCCGACGTTGC (Met1 forward),

GAT***GAATTC***TCAATCCATGGCAGCCAGCTG (Asp142 reverse),

GAT***GAATTC***TCAACCAAGGGTTAATTCTTCAAAC (Gly99 reverse) and

GAT***GGATCC***AAGAAGCAGTTTGAAGAATTAAC (Lys90 forward).

GST-fusion proteins were verified by SDS-PAGE and Coomassie staining and their concentration was determined by Bradford protein-assay.

### Solid Phase Assay

Bt-RGDS binding to recombinant GST-fusion proteins was evaluated by solid-phase assay (SPA) as previously described [46] with modifications. Briefly, microtiter plates (Costar, Cambridge, MA) were coated with 175 nM (100 μl/well) recombinant GST-fusion proteins diluted in AC7.5 buffer for 4 h at RT. Wells were blocked in 30 mg/ml BSA (300 μL/well) overnight at 4°C, washed and incubated for 4 h at RT with increasing doses of bt-RGDS (from 1.75 to 1.75 mM), in the presence or in the absence of an excess of not-biotinylated RGDS, as specific competitor. After washing four times and incubation for 1 h at RT with 100 μl/well Vectastain-ABC Reagent (Vector Laboratories, Burlingame, CA), wells were stained with the ELISA Amplification System (Invitrogen, Carlsbad, CA) according to manufacturer's instructions and absorption at 495 nm (*A*_495_) was determined. Specific binding was computed by subtracting nonspecific from total binding at each concentration. Curve fit was carried out according to the following one-site specific bind equation (GraphPad Prism-4 software):(1)

where X is ligand concentration, Y is the specific binding, Bmax is the maximum specific binding in the same units as Y, Kd is the equilibrium binding constant, in the same units as X.

### siRNA interference assays

SK-MEL-110 were seeded in 6-well-plates on collagen-IV (1 × 10^5 ^per well). Twenty-four hours later cells were transfected with human survivin siRNA (100 nM) or control siRNA-A (Santa Cruz Biotechnology) using Lipofectamine Plus Reagent according to manufacturer's instructions. SiGLO Lamin A/C siRNA (Dharmacon-RNAi Technologies) was used as control of transfection efficiency. After 72 h silencing, cells were treated for 48 h with FGF-2 (10 ng/ml) in the presence or in the absence of RGDS (500 μg/ml). Cells were counted using hemacytometer. Silencing of survivin protein was confirmed by western blotting.

### Statistics

Data were expressed as mean ± S.D. Student's two-tailed *t *test was performed and p ≤ 0.05 was considered statistically significant.

## Abbreviations

ECM: extracellular matrix; RGDS: Arg-Gly-Asp-Ser; RGES: Arg-Gly-Glu-Ser; RT: room temperature; FCS: Fetal calf serum; BSA: bovine serum albumin; HA: haemoagglutinin; siRNA: small interference RNA; FGF-2: human Fibroblast Growth Factor-2; bt-RGDS: biotinylated-RGDS; bt-RGES: biotinylated-RGES.

## Competing interests

The authors declare that they have no competing interests.

## Authors' contributions

MS Aguzzi: performed experiments, participated in data interpretation and manuscript writing; P. Fortugno: performed plasmid construction and purification, recombinant protein preparation and data interpretation; C. Giampietri: performed internalization experiments; G. Ragone performed internalization experiments; M.C. Capogrossi: participated in study coordination and data interpretation; A. Facchiano performed study supervision, data discussion and manuscript writing. All authors read and approved the final manuscript.

## Supplementary Material

Additional file 1 **RGDS effect on cell cycle in SK-MEL-110**. Cells were treated with FGF-2 (10 ng/ml) in the presence or in the absence of RGDS (500 μg/ml). RGDS treatment interferes with cell cycle. A representative histogram of three independent experiments was reported. Click here for file

Additional file 2 **RGDS internalization in HUVEC cells and SK-MEL-110**. RGDS internalization in HUVEC and SK-MEL-110 was measured by FACS. Cells were treated for 6 and 24 h with biotinylated-RGDS; internalization was revealed by PE-avidin and measured by FACS analysis. Three independent experiments were carried out. Click here for file

Additional file 3 **Survivin expression in HUVEC, melanocytes and SK-MEL-110**. Human epidermal melanocytes NHEM-neo (Lonza) were grown in MBM-2 supplemented with MGM-4 SingleQuots (CaCl2, FGF-2, PMA, rh-Insulin, Hydrocortisone, BPE, FBS and Gentamicin/Amphotericin-B) (Lonza). Human umbilical vein endothelial cells (HUVECs; Lonza) were maintained in EBM-2 medium (Lonza) supplemented with endothelial growth medium 2 (EGM-2) kit (FCS, hydrocortisone, hFGF-B, VEGF, R3-IGF-1, ascorbic acid, hEGF, GA-1000, heparin), according to manufacturer's instructions. Cells were cultured at 37°C in a 5% CO_2 _atmosphere. Survivin expression in three different cell types (HUVEC, NHEM-Neo or SK-MEL-110) was examined by western blotting. β-actin was used as control of equal loading. One representative experiment was reported, while the quantification refers to 3 different experiments. Click here for file
