# Supplementary figures and images for: Intracellular targets of RGDS peptide in melanoma cells
Source: Mol Cancer. 2010 Apr 22;9:84. doi: 10.1186/1476-4598-9-84 (PMC2867821; doi:10.1186/1476-4598-9-84)

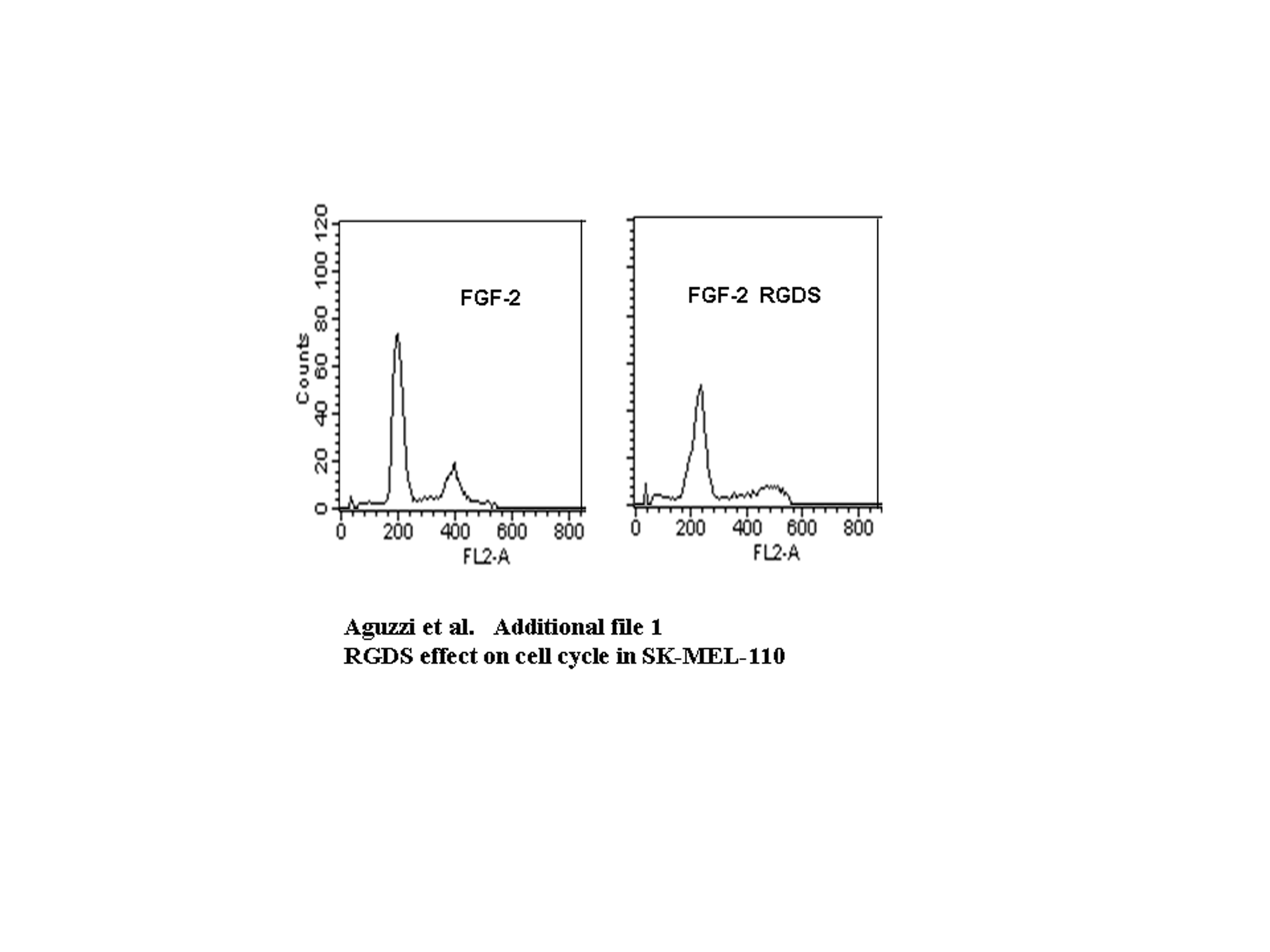

Supplement: Additional file 1 — RGDS effect on cell cycle in SK-MEL-110. Cells were treated with FGF-2 (10 ng/ml) in the presence or in the absence of RGDS (500 μg/ml). RGDS treatment interferes with cell cycle. A representative histogram of three independent experiments was reported. [file 1476-4598-9-84-S1.JPEG]

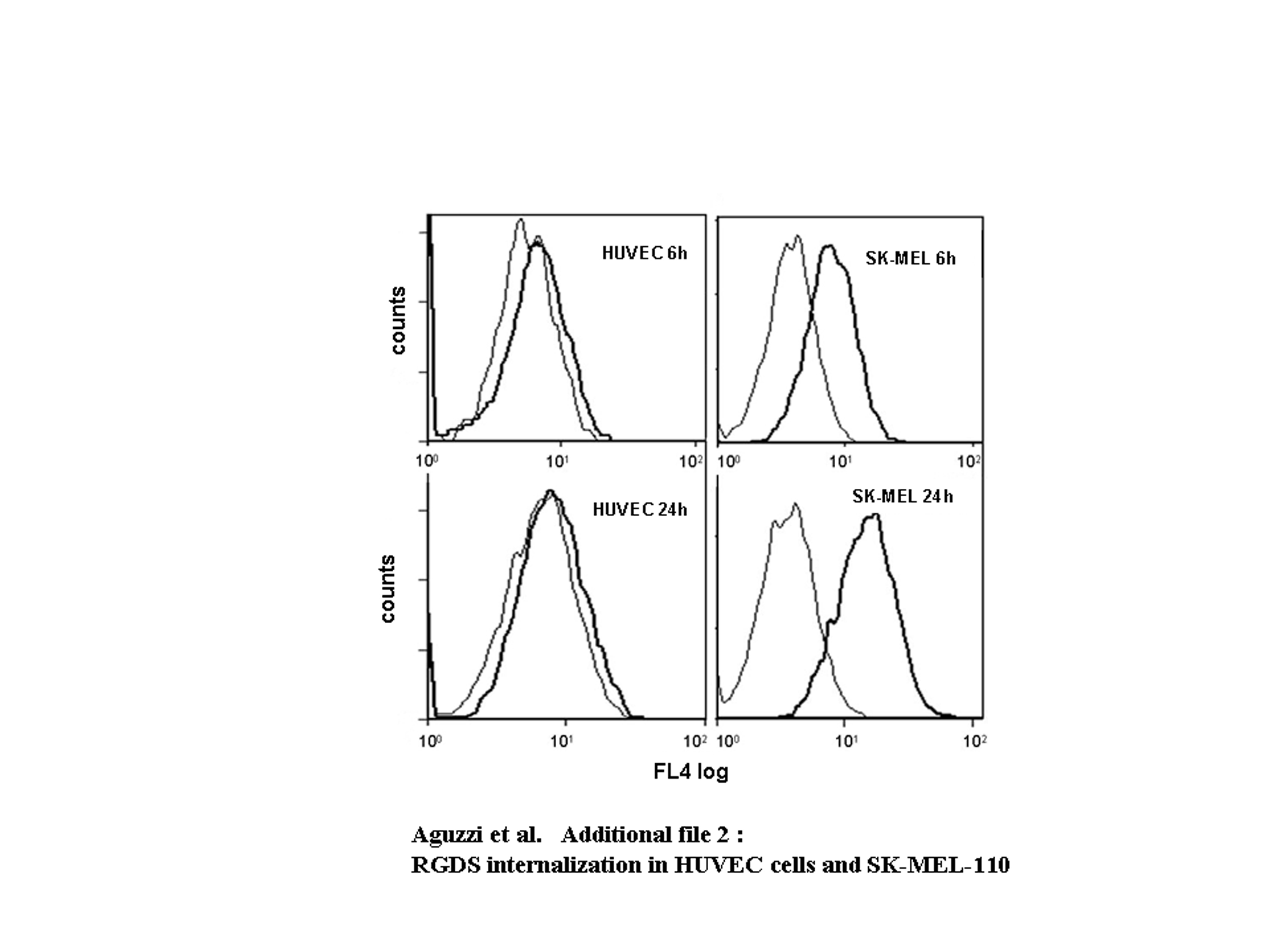

Supplement: Additional file 2 — RGDS internalization in HUVEC cells and SK-MEL-110. RGDS internalization in HUVEC and SK-MEL-110 was measured by FACS. Cells were treated for 6 and 24 h with biotinylated-RGDS; internalization was revealed by PE-avidin and measured by FACS analysis. Three independent experiments were carried out. [file 1476-4598-9-84-S2.JPEG]

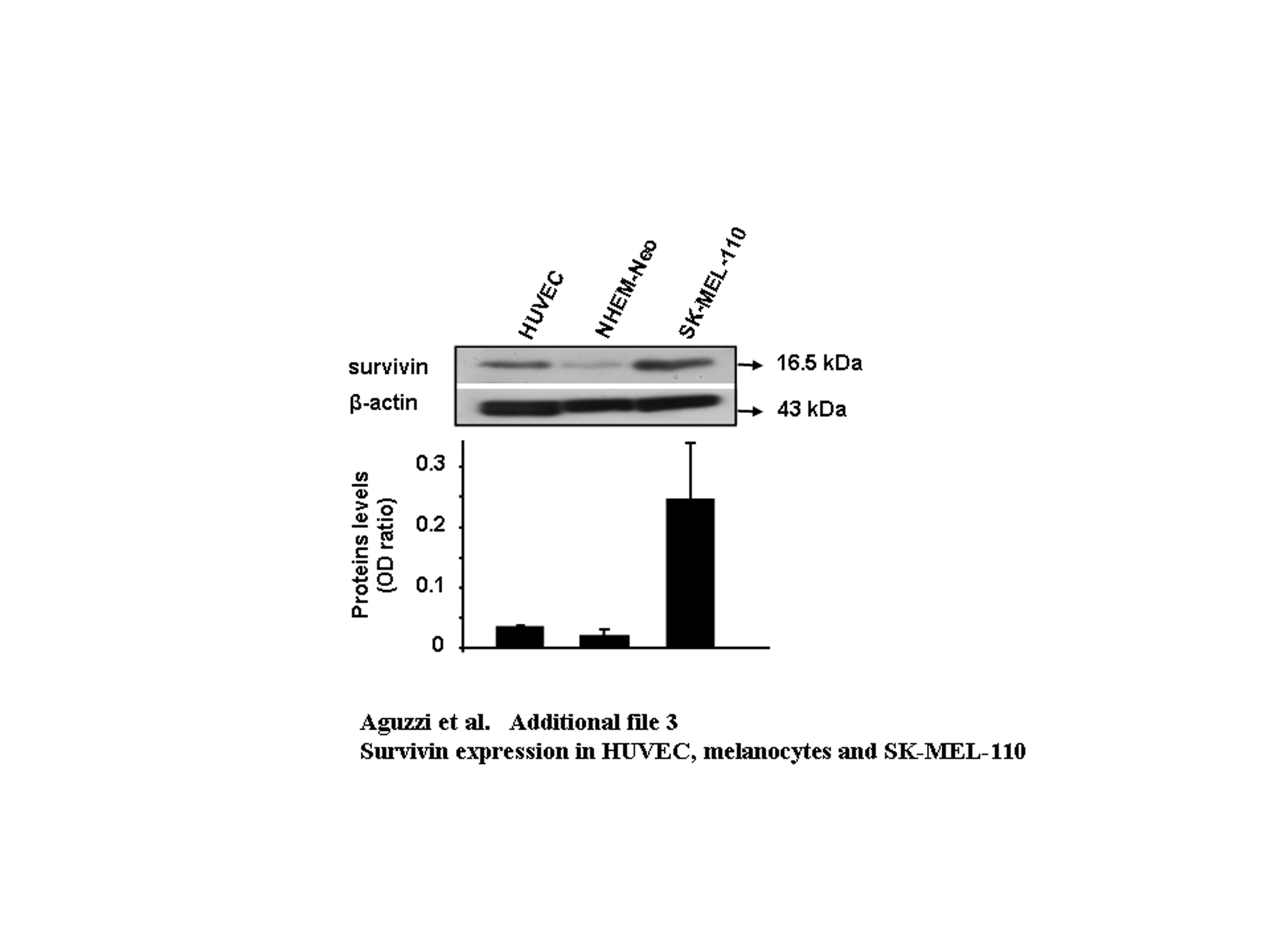

Supplement: Additional file 3 — Survivin expression in HUVEC, melanocytes and SK-MEL-110. Human epidermal melanocytes NHEM-neo (Lonza) were grown in MBM-2 supplemented with MGM-4 SingleQuots (CaCl2, FGF-2, PMA, rh-Insulin, Hydrocortisone, BPE, FBS and Gentamicin/Amphotericin-B) (Lonza). Human umbilical vein endothelial cells (HUVECs; Lonza) were maintained in EBM-2 medium (Lonza) supplemented with endothelial growth medium 2 (EGM-2) kit (FCS, hydrocortisone, hFGF-B, VEGF, R3-IGF-1, ascorbic acid, hEGF, GA-1000, heparin), according to manufacturer's instructions. Cells were cultured at 37°C in a 5% CO2 atmosphere. Survivin expression in three different cell types (HUVEC, NHEM-Neo or SK-MEL-110) was examined by western blotting. β-actin was used as control of equal loading. One representative experiment was reported, while the quantification refers to 3 different experiments. [file 1476-4598-9-84-S3.JPEG]
